# The changing role of family income in mental health from childhood to adolescence: findings from a UK longitudinal study

**DOI:** 10.1186/s13690-025-01702-4

**Published:** 2025-09-01

**Authors:** Murong Yang, Mara Violato, Claire Carson

**Affiliations:** 1 Nuffield Department of Primary Care Health Sciences, University of Oxford, Radcliffe Observatory Quarter, Woodstock Road, Oxford, OX2 6GG UK; 2 Nuffield Department of Population Health, University of Oxford, Old Road Campus, Oxford, OX3 7LF UK

**Keywords:** Child mental health, Income gradient, Health inequalities, Internalising problems, Externalising problems

## Abstract

**Background:**

Childhood and adolescence are critical periods of emotional and psychological development, during which socioeconomic factors such as family income may have varying effects on mental health. While previous research suggests that children from lower-income families tend to have poorer mental health outcomes, it is unclear how this association changes with age. Understanding these patterns is essential for designing interventions that target the most vulnerable periods in development. This study investigates the relationship between family income and mental health across childhood and adolescence in the UK, identifying potential age-related changes in the strength of this association.

**Methods:**

A sample of 5667 children from the UK Millennium Cohort Study was followed from ages 3 to 17 years. Overall mental health problems, internalising and externalising problems were measured using the Strengths and Difficulties Questionnaire. Family income was assessed using inflation-adjusted annual family income, adjusted for family size and composition, and a binary indicator of poverty status. Panel data linear fixed-effects models were used to control for unobserved heterogeneity.

**Results:**

Higher family income was associated with better child mental health, but the magnitude of the effect varied with age. After adjustment for confounders, lower income predicted poorer overall mental health at ages 11 and 14 years, with the strongest association observed at 14. Weak association was found at ages 3, 5, 7, and 17. A similar trend was observed for externalising problems, while the income protective effect on internalising problems showed a significant increase with age. No substantial sex differences were observed in these associations.

**Conclusions:**

Child mental health-income gradient exists in the UK and varies with age, being stronger in adolescence than childhood, and for internalising symptoms. Family income interventions/redistributions in early adolescence are more likely to reduce mental health problems in young people.

**Supplementary Information:**

The online version contains supplementary material available at 10.1186/s13690-025-01702-4.

**Table:** 

**Text box 1. Contributions to the literature**
• This study conducts a longitudinal analysis from early childhood to adolescence, covering one of the longest study periods in the literature on family income and child mental health.
• Using a panel data analytical approach, this study examines the age-specific association between family income and child mental health while controlling for unobserved heterogeneity.
• The findings offer evidence to inform policies to support economically disadvantaged families, highlighting the importance of intervention timing in reducing child mental health inequalities.

## Introduction

Mental health problems are increasingly prevalent among children and adolescents in the UK [1] and worldwide [2]. Poor mental health in childhood has detrimental effects on education attainment [3, 4] and, later in life, performance in the labour market [5], which in turn may decrease family income and cause economic costs in adulthood such as reduced ability to work and earnings [6, 7]. Growing child mental health difficulties have also increased the burden on the UK public healthcare system [8].

The determinants of mental health and well-being in children and adolescents are complex and multifactorial. Among potential factors impacting child mental health, socioeconomic status (SES), which reflects family income, parental education and occupational status, has raised significant attention. Children from low-income families and those with parents who are less educated, from unskilled occupations, or experience unemployment face a heightened risk of having mental health problems [9–14]. While there are different indicators of SES, family income is considered an important factor for child development. Lower-income families often have fewer resources to invest in their children, such as better childcare and spending quality time in activities with their children [15]. Poverty is also associated with increased family stress and poorer parental physical and mental health (Yeung et al., 2002), which may negatively affect parenting behaviours. These factors, in turn, may has a negative effect on their children’s outcomes [16, 17]. Family income also holds particular importance for policymaking, with income support interventions - such as cash transfer programs and tax credits - being widely implemented [18].

The association between lower family income and poor child mental health is often referred to as the ‘income gradient in child mental health’ [19] and this gradient has been investigated in previous empirical studies. However, most studies have conducted a cross-sectional analysis by using one specific age group in either childhood or adolescence and have yielded inconsistent findings. The gradient was observed in adolescence (10–19 years old) using data from the UK [20], the US [21] and Sweden [22]. However, evidence in early childhood (0–5 years) and middle childhood (6–9 years) is limited and ambiguous. Violato, Petrou [23] found a significant association between higher family income and better mental health at ages 3 and 5 years, separately, using data from the UK Millennium Cohort Study. Washbrook [24] and Yamaoka, Isumi [25] found no evidence of an association at 7 and 9 years old in the UK, and at 6 and 7 years old in Japan, respectively. The use of a specific age group in these studies makes it difficult to compare the income gradient for the same group of children across different developmental stages.

Using a repeated cross-sectional data from the UK Millennium Cohort Study, Yang, Carson [26] found that the income gradient was steeper during adolescence than in childhood. However, this study, like the studies mentioned above, followed a cross-sectional design, which fails to account for the dynamic change of the same individual over time. The cross-sectional relationship between income and child mental health can be affected by unobserved heterogeneity, which occurs whenever potentially unobserved differences between children are correlated with the explanatory variables and child mental health outcomes in the empirical models. Unobserved heterogeneity may lead to biased and inconsistent estimates of the income coefficient [27], but this methodological limitation can be minimised by utilising a panel data analysis approach.

Previous panel data studies commonly assumed that the relationship between family income and child mental health remains constant as children grow up, yielding the same income gradient across all age groups. These studies either found no effect [28] or a negative effect of family income on child mental health problems [29, 30], while the true income effect at a specific developmental stage may be diluted if the income gradient varies between different age groups. Research has shown that exposures of interest, such as low income, may have a stronger effect during particular stages of child development [31, 32]. However, few studies have examined whether the income gradient varies at different stages of child development using a panel data approach.

The income gradient may vary between internalising and externalising problems, which are treated as two broad categories of mental health problems [33]. Internalising problems can be defined as internally focused problems, reflecting the emotional state of mental well-being, such as anxiety and depression. Externalising problems include externally focused behavioural problems, such as hyperactivity and aggression, which may cause conflicts in the external environment and potentially affect others [34–36]. The income gradient in internalising and externalising problems has been recognised in previous studies, but there is still limited evidence on whether it varies with age. Using longitudinal data, Najman, Clavarino [37] found that compared with poverty in early childhood, poverty in adolescence was an important risk factor for depression and anxiety at 14 years old in Australia. However, it is unclear whether the same holds for externalising problems, and also for children in the UK.

In addition, the prevalence of internalising and externalising problems has been shown to differ by sex, with internalising problems becoming more common for girls than boys during adolescence [38]. Evidence suggests that these sex differences may be linked with family income. For example, in the US, living in poverty was found as a risk factor for externalising problems among girls in early childhood [39]. Similarly, consistent exposure to poverty was found to predict internalising problems in adolescent girls in Australia, while these associations were not observed for boys [40]. However, a recent study using UK Millennium Cohort Study found no sex differences in the association between family income at 9 months and child internalising and externalising problems in childhood and adolescence [41]. These mixed findings highlight the need for further research to examine whether the income gradient in child mental health varies by sex, particularly across different developmental stages.

This study aims to examine the variation in income gradient in child mental health from early childhood to adolescence, using data from the UK Millennium Cohort Study. We utilised panel data analysis to investigate the potential age-specific income gradient in child mental health, using a consistent UK cohort followed up from 9 months to 17 years of age. Our study makes several contributions to the existing literature on the relationship between family income and child mental health. First, we examined the age-specific income gradient in child mental health that is rarely presented by previous literature. Because of the panel nature of the dataset, we could follow the same child and use consistent measures over time. Furthermore, we tested the income gradient both in overall child mental health and its internalising and externalising aspects, and examined whether this gradient differed by child sex for each domain. In addition to family income, we also used poverty as an alternative indicator of financial status to capture the impact of extremely low income on child mental health. The findings from this study may inform income-related policy intervention and/or re-allocation strategies that target reducing child mental health problems.

## Methods

### Data and study population

Data were from the UK Millennium Cohort Study (MCS), a nationally representative longitudinal study of 18,818 children (from 18,552 families) born in the UK between 2000 and 2002 [42]. The MCS uses a stratified sampling design, oversampled children living in devolved nations, in areas with a high proportion of children living in poverty, and with high ethnic minority populations in England [42]. In our study, we applied the MCS sample design weight, which corrects for the unequal probabilities of sample selection caused by stratified cluster sampling design [42].

The first MCS survey was administered when children were 9 months old, and the subsequent surveys when children were aged 3, 5, 7, 11, 14, and 17 years, with the age 23 survey not available for analysis yet. Families recruited in the study may miss one or more surveys. The sample size decreased as children grew older, with 10,625 families (57.3% of the original number) participating in the seventh survey [43].

MCS was conducted by trained interviewers using face-to-face interviews, along with online questionnaires. It captures detailed information about each family, including child and parental health, parental socioeconomic status, pregnancy-related factors, and other family circumstances and lifestyles [42]. This paper used data from all available surveys when children were aged 9 months, 3, 5, 7, 11, 14 and 17 years [44–52]. To construct a balanced panel dataset that followed the same children across surveys, the study population was restricted to singletons who participated in all seven surveys. To improve consistency in reporting, the study population was further restricted to children for whom the Strengths and Difficulties Questionnaire - the tool for measuring child mental health outcomes - was answered by the biological mother in all surveys.

### Variables

#### Child mental health problems

Child mental health problems were assessed using the Strengths and Difficulties Questionnaire (SDQ) when children were aged 3, 5, 7, 11, 14 and 17 years old. The SDQ includes 25 items which are grouped into 5 subscales: emotional problems, peer problems, conduct problems, hyperactivity and prosocial behaviour, with each subscale including 5 items. SDQ is a validated screening tool to measure child emotional and behavioural problems [53–55]. The primary outcome in this study was mother-reported *overall mental health problems* measured by the Total Difficulties Score (TDS), calculated by adding all the SDQ subscales except for prosocial behaviour. The secondary outcomes were mother-reported *internalising* and *externalising problems*, measured by adding the emotional and peer problems subscales for first, and conduct problems and hyperactivity subscales for the latter, respectively. The broader internalising and externalising scales are a better indicator of difficulties than the five separate SDQ subscales in the general population such as this study [33, 56]. The TDS ranges from 0 to 40, while both internalising and externalising problems scores range from 0 to 20. A higher score indicates more mental health problems. To aid direct comparison in the analyses, all three scores were standardised, with mean equal to 0 and standard deviation equal to 1.

#### Family income

MCS respondents reported their annual family income in each survey. We first derived equivalised annual income using the modified Organisation for Economic Co-operation and Development equivalence scale to adjust for family size and composition. Each family member was assigned a weight: 0.67 for the first adult, 0.33 for the spouse and each dependent child aged between 14 and 18 years old, and 0.20 for each child aged under 14 years old [57]. The equivalence scale for a family was a sum of these weights. Equivalised family income was calculated by dividing the reported annual family income by this scale. To account for inflation, we adjusted equivalised family income collected from the first survey (age 9 months, conducted in 2001) to the sixth survey (age 14 years, conducted in 2015) using the Average Weekly Earnings Index [58], with 2018, the year of the seventh survey (age 17 years), as the base year. The resulting inflation-adjusted, equivalised family income for each survey was used to represent transitory income, which captures short-term income measured at a given point in time.

The key independent variable in this study was *lagged transitory income*, which was measured by transitory income in the previous survey. Using lagged income can reduce the bias due to the potential impact of child mental health problems on family income if assessed at the same point in time, reducing the risk of reverse causation. Poverty was used as an alternative independent variable. We used *lagged transitory poverty*, measured by poverty in the previous survey. The poverty variable was derived by the MCS team and defined as family income below 60% of the national median household equivalised income [43].

#### Other explanatory variables

The explanatory variables included in our analyses were grouped as follows (Table 1): initial endowments, pregnancy-related factors, child characteristics, family socioeconomic factors, and the wave variable. ‘Initial endowments’ included variables that are determined before birth. The rationale for including these variables is further explained in the Empirical Strategy section. Variables reflecting pregnancy-related factors, child characteristics, and family socioeconomic factors were considered as potential confounding factors, as these variables are associated with both income and child mental health outcomes, and they do not lie on the causal pathway from family income to child mental health. A wave control variable was generated and included in the panel data models to capture the specific survey the observations belonged to (1 = Wave 1 or the first survey, 2 = Wave 2, …, 7 = Wave 7). Variable details are reported in Appendix Table A1.

**Table 1:** Variable list

Variable category	Type	Variable name
Initial endowments	Time-varying	Child’s age
	Time-invariant	Child’s sex, ethnicity, gestational age, firstborn
Confounders	Time-varying	*Child characteristics*: Child’s limiting physical longstanding illness, child’s body mass index (BMI) *Family socioeconomic characteristics*: Lone parent, change in family structure, maternal education
	Time-invariant	*Pregnancy-related factors*: Maternal age at childbirth, maternal smoking during pregnancy, maternal alcohol consumption during pregnancy, breastfeeding duration
Wave control	Time-varying	Wave variable

### Empirical strategy

#### Empirical model

The empirical model draws on the framework of the health production function [59] and the extended child health production function [60]. These models predicate that children are born with initial endowments, and their health is the product of parental and other inputs. To reduce the omitted variable bias caused by individual-invariant but time-varying factors, such as unobserved age-specific factors, the wave variable illustrated above was added to the model. Family income and wave interaction term was also added to the model to capture the potential variation in the ‘child mental health – family income gradient’ by age group (or wave of survey data collection). The empirical model can be written as:

1 $$\begin{array}{l}\:{MH}_{it}=\\\beta\:ln{\left(Y\right)}_{it}+{\alpha\:}_{t}{wave}_{t}+{\tau\:}_{t}ln{\left(Y\right)}_{it}*{wave}_{t}+{\varvec{X}}_{\varvec{i}\varvec{t}}\varvec{\delta\:}+{a}_{i}+{u}_{it}\end{array}$$

where *MH*_*it*_ is child *i*’s mental health status at time *t*; *Y* is family income, operationalised in its logarithmic form $\:\text{l}\text{n}{Y}_{it}$, and $\:{\varvec{X}}_{\varvec{i}\varvec{t}}$ is a set of explanatory variables other than income and can be written as follows:

2 $$\:{\varvec{X}}_{\varvec{i}\varvec{t}}={{{\gamma\:}_{I}I}_{i}+\gamma\:}_{c}{C}_{it}$$

which includes initial endowments or initial health stock *I* that do not change with time, and a set of confounding factors *C*. $\:{a}_{i}$ from Eq. (1) is an unobserved effect for child *i*, also known as individual heterogeneity, that does not change over time; $\:{u}_{it}$ is a time-varying error that changes over time [27]. The overall income association with child mental health in Wave *t* (i.e. marginal effect of income at Wave *t*) equals $\:\beta\:+{\tau\:}_{t}$.

#### Estimation methods and model selection procedure

We used panel data methods to estimate the association between family income and child mental health across survey waves. Specifically, we applied both fixed-effects (FE) and random-effects (RE) estimation methods to reduce the omitted variable bias caused by unobserved heterogeneity [61]. The FE approach controls for time-invariant individual characteristics by using with-in child variation, while the RE approach assumes that the unobserved individual effect is not correlated with the explanatory variables. The Hausman test was then used to identify the most appropriate approach [62].

After establishing the preferred estimation approach, we proceeded with model selection for each mental health outcome to establish which of the explanatory variables $\:{\varvec{X}}_{\varvec{i}\varvec{t}}$ were to be included in our final panel data models. Initial endowments were included on an a priori basis. Child age was excluded because it was collinear with the wave variable $\:{wave}_{t}$. Then, each $\:{X}_{it}$ variable reflecting ‘pregnancy-related factors’, ‘child characteristics’ and ‘family socioeconomic factors’ in Table 1 were added into the models sequentially. Forward selection was used and model fit was assessed at each step using a Wald test until the preferred model specification was established. We will refer to this preferred model specification hereafter as the ‘*fully adjusted model*’. The age-specific income gradient was examined by conducting an F-test to examine whether the overall income coefficients were statistically different across survey ages. The same model selection procedure was applied separately for boys and girls, and sex differences in the age-specific income gradient was examined using a two-sided z-test.

#### Missing data

We examined the patterns of missing data within and across surveys. To account for missing cases where a child did not participate in certain surveys, i.e. unit nonresponse, we used inverse probability weights generated by the MCS team [42]. For missing cases due to item nonresponse, when cohort members who participated in the survey failed to answer all the questions, those missing values were filled in by multiple imputation using chained equations [63]. The imputation model included all variables that were used in the subsequent analysis, as well as auxiliary variables such as child-parent relationship at age 3, childcare arrangements at 9 months and 3 years, maternal mental and physical health, housing tenure, and parenting activities from 9 months to 17 years. Thirty imputed datasets were generated and combined using Rubin’s rules [64].

#### Sensitivity analysis

We conducted multiple imputation to address missing data from both item nonresponse and unit nonresponse [65], allowing us to expand the study population to include all singletons who participated in the initial MCS sample at 9 months. The imputation model was consistent with the main analysis, and seventy imputed datasets were generated and combined using Rubin’s rules. The main analysis models were then replicated using the expanded study population. Sample weights at 9 months, provided by the MCS team, were applied in the models to adjust for sampling errors in the initial sample [66].

Another sensitivity analysis was conducted to explore potential selection bias due to missing data. The models presented in the main analysis were replicated using a population that had complete data for all variables (complete case analysis).

All analyses were conducted using Stata/SE 16.0 [67].

## Results

### Descriptive statistics

Of the 5667 singleton children in the analysis, about half (48.9%) were male, and 8% were of minoritised ethnicity (Table 2). The mean TDS was highest at 3 years old, with a value of 8.9. It decreased to 6.7 and 6.9 at 5 and 7 years old, respectively, but increased to 7.2–7.5 in late childhood and adolescence (ages 11, 14, and 17 years). The mean internalising symptom score, which ranged from 2.6 to 3.7, was lower in early childhood and increased as children grew older, with the highest score of 3.7 observed at age 17. Externalising problems had the highest mean score of 6.3 at age 3, and the score decreased gradually to 3.4 at age 17. For other variables see Appendix Table A2.

Children who have missing data due to item non-response were more likely to be of minoritised ethnicity, and more likely to be born to younger mothers with lower educational attainment, who were more likely to smoke in pregnancy and less likely to breastfeed for the recommended duration (Appendix Table A3).

### The association between family income and child mental health

When comparing the FE and RE estimators using the Hausman test, the test strongly rejected (*p* < 0.001, Appendix Table A4) the null hypothesis that the unobserved effect is not correlated with the explanatory factors, which indicates that FE was more appropriate than RE in this study. Thus, the relationship between family income and child mental health was examined using the FE estimator in the following analyses.

**Table 2:** Descriptive characteristics of the study population

Variables	3 years	5 years	7 years	11 years	14 years	17 years
**Child mental health**						
Total Difficulties Score-Mean (SD)	8.9 (4.9)	6.7 (4.7)	6.9 (5.1)	7.2 (5.7)	7.5 (5.8)	7.0 (5.8)
Internalising problems-Mean (SD)	2.6 (2.3)	2.3 (2.4)	2.5 (2.6)	3.1 (3.1)	3.6 (3.4)	3.7 (3.5)
Externalising problems-Mean (SD)	6.3 (3.6)	4.4 (3.3)	4.4 (3.4)	4.1 (3.4)	4.0 (3.4)	3.4 (3.2)
**Family income**						
Lagged transitory income (£)-Mean (SD)	30,893	29,742	28,379	28,685	28,598	27,131
	(17840)	(17429)	(15650)	(15106)	(10193)	(9837)
Lagged transitory poverty-n (%)	1411 (18.7)	1388 (18.9)	1400 (19.4)	1269 (17.3)	901 (10.8)	1179 (14.6)
**Initial endowments**						
Age, years-Mean (SD)	3 (0.2)	5 (0.2)	7 (0.2)	11 (0.3)	14 (0.3)	17 (0.3)
Male-n (%)	2737 (48.9)	-	-	-	-	-
Minority ethnic group-n (%)	701 (8.0)	-	-	-	-	-
Preterm (< 37 weeks gestation)-n (%)	396 (7.1)	-	-	-	-	-
Firstborn-n (%)	2893 (51.8)	-	-	-	-	-

Notes: *N* = 5667; unweighted counts (n) and survey-weighted mean, standard deviation (SD) and proportions (%) reported; transitory income adjusted for inflation and family size; - baseline variable which remains the same across all surveys

#### Overall mental health problems

**Fig. 1:**
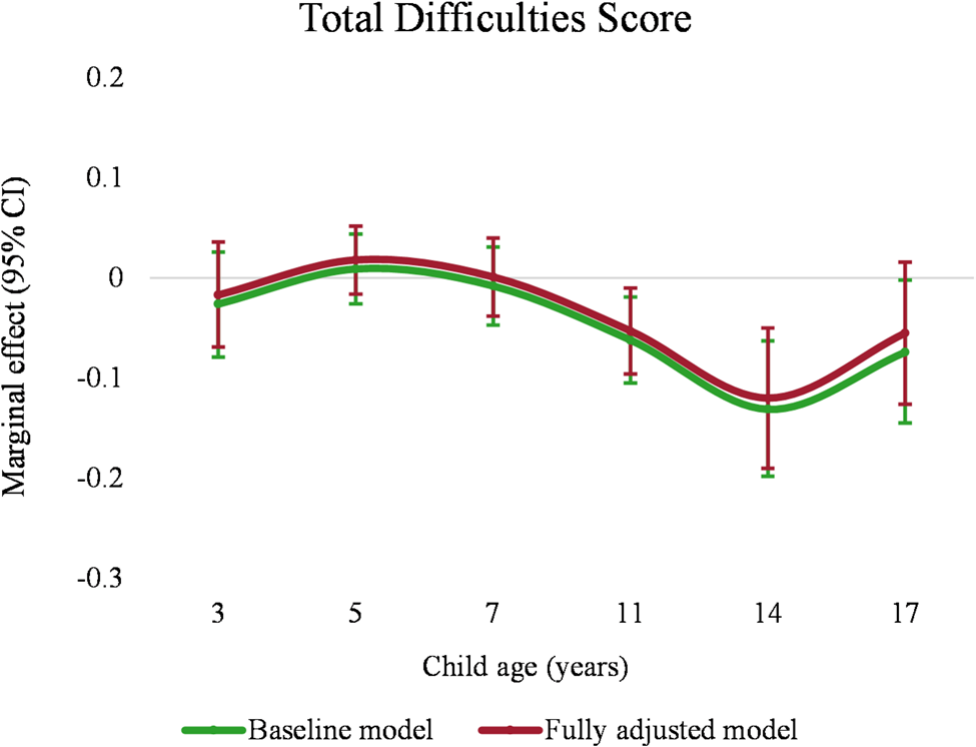
Marginal effects of income on child overall mental health problems

Table 3 shows the results of the association between family income and child overall mental health problems using two model specifications, i.e. the baseline model controlling for wave and wave and income interaction (S1), and the fully adjusted model further controlling for initial endowments and confounding factors (S2) (see Appendix Table A5 for full regression results). Due to the nature of fixed-effects estimation, all the time-invariant variables were excluded from the models. The overall income association with child overall mental health problems in each age group (i.e. the marginal effect of income) was derived by combining the income coefficient with the corresponding interaction terms from Table 3, as outlined in the Methods section. This result is reported in Fig. 1 and Table A6. Fig. 1 shows that the trajectory of the association between family income and child overall mental health problems across age groups was consistent in two model specifications (S1 and S2). A significant association between lower family income and worse overall mental health problems was found at 11 and 14 years. The magnitude of the marginal effect was the largest at 14 years, where a 1% increase in lagged transitory income was associated with a decrease of around 0.1 SD in the Total Difficulties Score. However, the magnitude of the marginal effect was smaller in childhood compared with adolescence and not statistically significant at the 5% level. An F-test showed that the marginal effects from 3 to 17 years old were significantly different from each other (*p* < 0.01).

**Table 3:** The association between family income and child overall mental health problems (TDS)

		S1	S2
Lagged transitory income		-0.026	-0.017
		(0.027)	(0.027)
**Survey wave (child age)**			
Wave 2 (3 years) #		-	-
Wave 3 (5 years)		-0.760***	-0.763***
		(0.240)	(0.241)
Wave 4 (7 years)		-0.560**	-0.567**
		(0.238)	(0.239)
Wave 5 (11 years)		0.046	0.036
		(0.275)	(0.276)
Wave 6 (14 years)		0.812*	0.780*
		(0.419)	(0.430)
Wave 7 (17 years)		0.137	0.015
		(0.442)	(0.443)
Income × Wave 2 #		-	-
Income × Wave 3		0.035	0.035
		(0.024)	(0.024)
Income × Wave 4		0.018	0.019
		(0.023)	(0.023)
Income × Wave 5		-0.036	-0.036
		(0.027)	(0.027)
Income × Wave 6		-0.105**	-0.103**
		(0.041)	(0.042)
Income × Wave 7		-0.049	-0.038
		(0.043)	(0.043)
**Other controls**		No	Yes

Notes: # reference category; S1: baseline model, controls for wave and interaction between income and wave; S2: fully adjusted model, further controls for initial endowments and confounders *** *p* < 0.01, ** *p* < 0.05, * *p* < 0.1

#### Internalising and externalising problems

**Fig. 2:**
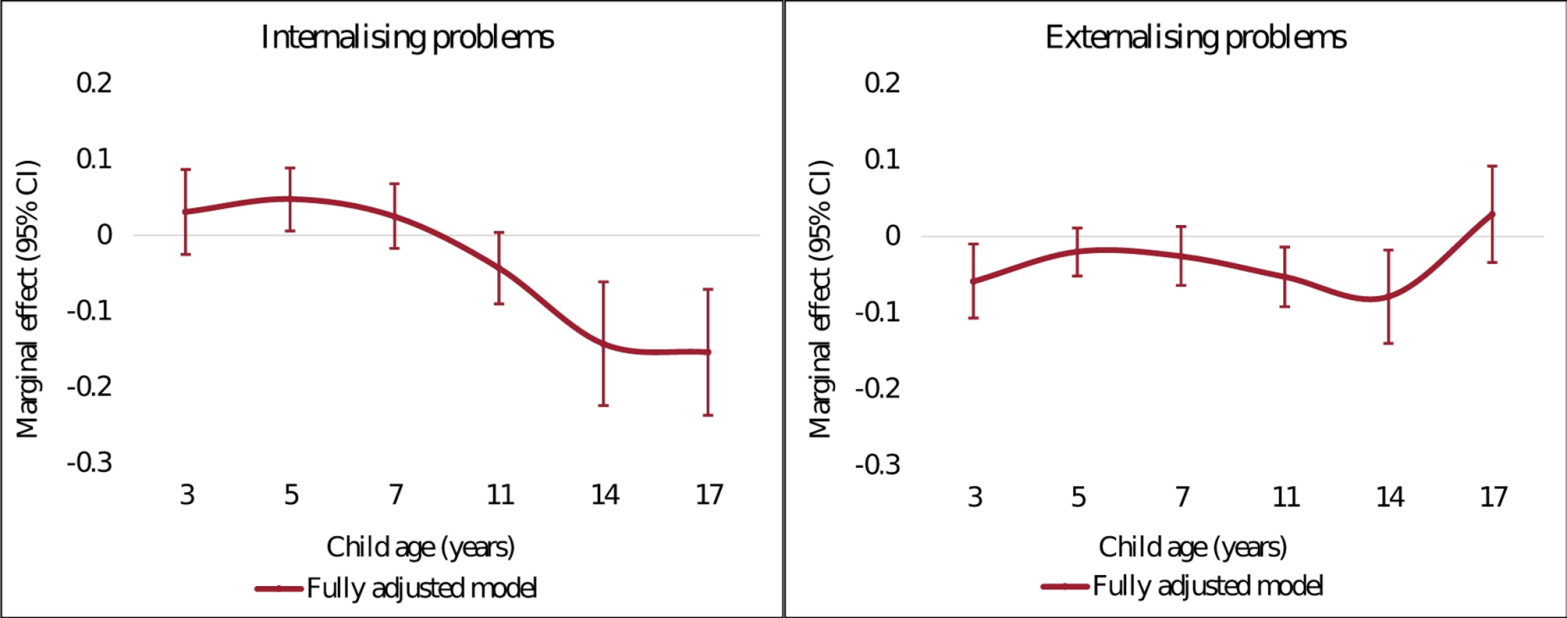
Marginal effects of income on internalising/externalising problems

Table 4 shows the results of the association between family income and internalising and externalising problems using two model specifications (S1-S2). The marginal effects of income on child mental health outcomes are reported in Fig. 2, which shows that the trajectory of the association between family income and child mental health over time differed for internalising and externalising problems (full regression results see Tables A7 and A8). In the fully adjusted model (S2), higher family income was strongly associated with fewer internalising problems in adolescence. The magnitude of the association, in absolute terms, increased from 11 to 17 years and was largest at 17 years, where a 1% increase in lagged transitory income produced a decrease of 0.15 SD in the internalising symptom scores (*p* < 0.01). The marginal effects were smaller in childhood. An association between lower family income and worse externalising problems was observed at ages 3, 11 and 14 years old and was largest at 14 years, with a 1% increase in income reducing the score by an average of 0.08 SD (*p* < 0.01). The F-test showed that the marginal effects from 3 to 17 years old were significantly different from each other for both internalising and externalising problems (*p* < 0.01). Point estimates and statistical significance were consistent between the baseline model and fully adjusted model (Appendix Figure A1 and Table A9).

**Table 4:** The association between family income and internalising/externalising problems

	Internalising			Externalising		
	S1		S2	S1		S2
Lagged transitory income	0.029		0.031	-0.064***		-0.059**
	(0.028)		(0.028)	(0.025)		(0.025)
**Survey wave (child age)**						
Wave 2 (3 years) #	-		-	-		-
Wave 3 (5 years)	-0.273		-0.286	-0.937***		-0.936***
	(0.227)		(0.229)	(0.254)		(0.254)
Wave 4 (7 years)	0.046		0.026	-0.900***		-0.882***
	(0.241)		(0.243)	(0.253)		(0.253)
Wave 5 (11 years)	0.953***		0.883***	-0.736***		-0.671**
	(0.299)		(0.298)	(0.261)		(0.261)
Wave 6 (14 years)	2.163***		2.075***	-0.581		-0.455
	(0.471)		(0.474)	(0.371)		(0.372)
Wave 7 (17 years)	2.441***		2.200***	-1.837***		-1.725***
	(0.494)		(0.491)	(0.384)		(0.384)
Income × Wave 2 #	-		-	-		-
Income × Wave 3	0.016		0.017	0.040		0.040
	(0.022)		(0.022)	(0.025)		(0.025)
Income × Wave 4	-0.008		-0.006	0.034		0.033
	(0.024)		(0.024)	(0.025)		(0.025)
Income × Wave 5	-0.080***		-0.074**	0.012		0.006
	(0.029)		(0.029)	(0.025)		(0.025)
Income × Wave 6	-0.182***		-0.174***	-0.008		-0.020
	(0.046)		(0.046)	(0.036)		(0.036)
Income × Wave 7	-0.206***		-0.185***	0.099***		0.088**
	(0.048)		(0.048)	(0.038)		(0.038)
**Other controls**	No		Yes	No		Yes

Notes: # reference category; S1: baseline model, controls for wave and interaction between income and wave; S2: fully adjusted model, further controls for initial endowments and confounders; *** *p* < 0.01, ** *p* < 0.05, * *p* < 0.1

### The association between poverty and child mental health

#### Overall mental health problems

**Fig. 3:**
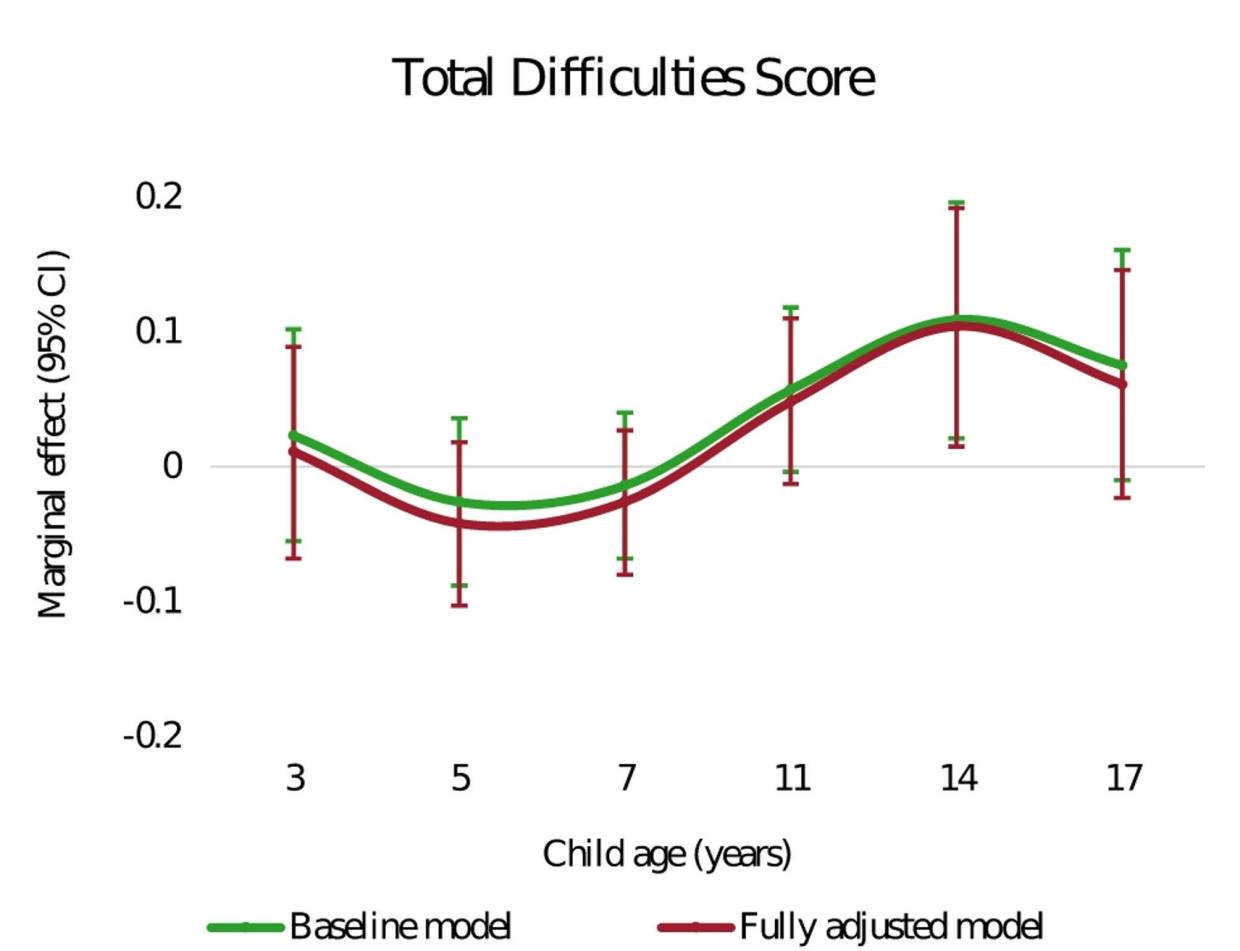
Marginal effects of poverty on child overall mental health problems

Fig. 3 (and Appendix Table A10) shows the marginal effects of poverty on child overall mental health problems in each age group. In the fully adjusted model (S2), when children were aged 3, 5, and 7 years, the associations were close to zero. When children grew older, i.e. at 11, 14 and 17 years, those who were living in poverty had worse mental health symptoms than children who were not. Consistent with the results from family income, the marginal effect of poverty was largest at 14 years, where living in poverty was associated with an increase of 0.1 SD of the TDS. For full regressions see Appendix Table A11.

#### Internalising and externalising problems

**Fig. 4:**
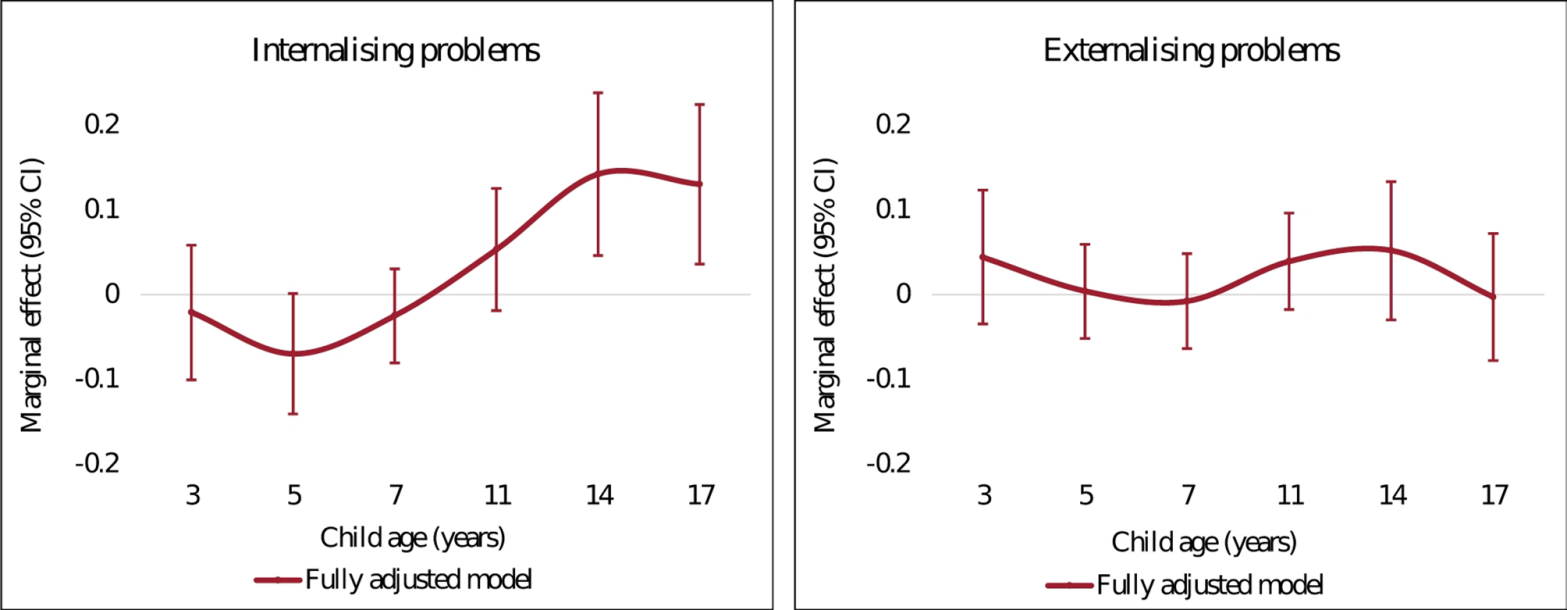
Marginal effects of poverty and internalising/externalising problems

In the fully adjusted model (S2), children living in poverty experienced worse internalising problems at 11, 14 and 17 years old (Fig. 4). Living in poverty was associated with an increase of around 0.13 SD of internalising symptom scores at 14 and 17 years old. The association for externalising problems was relatively small compared with internalising problems. The results from model specifications S1 (baseline model) were similar to the fully adjusted model S2 in terms of point estimates and statistical significance (Appendix Table A12). For full regressions results see Tables A13 and A14.

### Income gradient by child sex

**Fig. 5:**
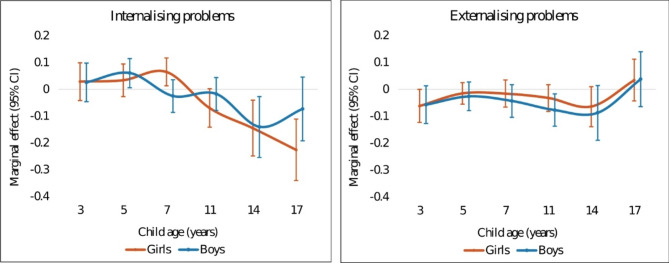
Marginal effects of income on internalising/externalising problems by child sex. Notes: fully adjusted model used; *n* = 2737 for boys and *n* = 2930 for girls

Fig. 5 shows the age-specific income gradient for internalising and externalising problems by child sex, based on the fully adjusted model. The point estimates for internalising problems suggested that the association for boys fluctuated but decreased from 14 to 17 years old, while the association for girls increased in magnitude over time and reached the largest at 17 years old, where a 1% increase in lagged transitory income was associated with a decrease of 0.23 SD in the child internalising symptom score (*p* < 0.01). The point estimates for girls were larger than boys in magnitude from 11 to 17 years, while no statistically significant sex difference was observed, except for 7 and 17 years old. For externalising problems, the point estimates were similar for boys and girls, and there was no strong evidence of a statistical difference between boys and girls at each time point. Full results are reported in Appendix Table A15.

### Sensitivity analysis

By using multiple imputation to fill in missing cases due to both item and unit non-response, the study population was expanded to 18,294 singleton children (8,879 girls and 9,415 boys), of whom 42.3% participated in all seven survey waves. The pattern of the income gradient in child mental health outcomes across age groups were consistent with the main analysis based on the smaller sample (*N* = 5,667), although the point estimate of the marginal effects of income at ages 14 and 17 years were slightly smaller for both TDS and internalising problems in the expanded sample (Figure A2). No sex differences were observed in the income gradient for internalising and externalising problems using the expanded sample (Figure A3).

Results from the complete case analysis on the association between lagged transitory income and TDS were in line with the main analysis using imputed data (Figure A4).

## Discussion

Using balanced panel data from seven successive surveys of the UK Millennium Cohort Study, this study shows that lower family income was associated with more adverse child mental health symptoms, but the magnitude of the association varied with age. After adjustment for confounding factors, lower income predicted poorer overall mental health at 11 and 14 years, with the largest association in magnitude observed at 14, while there was little evidence of an association at ages 3, 5, 7 and 17 years. A similar trend was observed for externalising problems, while in contrast, the income gradient in internalising problems increased from childhood to adolescence and reached the largest magnitude at 17 years. Results using poverty as the indicator of family financial position were consistent with those using family income and reinforced the importance of children not living in deprivation as a way of reducing child mental health problems. No substantial sex differences were observed in the income gradient for internalising and externalising problems.

The results presented in this study indicate that there is not always evidence of an association between family income and child mental health, and the income gradient in child mental health does not consistently increase or decrease over time, but may be larger in magnitude at specific developmental stages than others, with adolescence shown as the important period for both overall mental health problems, and internalising and externalising problems. This is consistent with findings from existing studies which used different age groups [20, 23, 24, 68]. The result that the relationship between family income and child mental health was strongest in adolescence, particularly for internalising problems, is consistent with the findings of large longitudinal studies in the UK [69] and US [70, 71].

The ‘age-specific’ income gradient in child mental health might be explained by the sensitive period assumption in the life-course model. This assumption states that there are certain periods in the life course when investment in outcomes, such as health, is more productive than at other times [72]. This also means that exposures of interest - such as low family income or poverty - have a stronger effect in a specific period of child development [31, 32]. Early childhood is conventionally recognised as a sensitive period for child development. This is because inputs provided during this time, including educational and financial support, can have a larger and longer impact than the same inputs provided later in life [72]. The early years have been identified as a sensitive period for improving cognitive outcomes such as mathematical and reading skills [32].

However, findings presented in this study suggest adolescence is also a sensitive period, particularly for mental health. Child mental health outcomes may be sensitive to parental inputs in adolescence because adolescence plays an important role in the formation of social and emotional habits closely linked to mental health [73]. Evidence shows that almost half (48.4%) of mental disorders start before age 18, with 14.5 being the most common age of onset for mental disorders globally [74]. Given the vulnerability of this period, exposure to low income or poverty during adolescence may result in worse mental health outcomes than exposure during childhood, potentially contributing to the stronger relationship between income and child mental health in adolescence. The findings from this study are in line with the UK study by Lai, Wickham [75] which suggested that poverty experienced in late childhood had a larger association with overall mental health in 14-year-olds compared to poverty experienced in early childhood.

The association between income and child mental health may be explained by other factors, as observed in previous studies. For example, positive early parenting and maternal depression have been shown to attenuate the income gradient in child internalising and externalising problems [26, 76]. Understanding these mediating pathways has important implications for designing targeted interventions to reduce the child mental health gap between the rich and poor.

### Strengths and limitations

A strength of this research is the longitudinal study that covered different child developmental stages. The study covered one of the longest periods of investigation that can be found in the existing empirical literature on family income and child mental health. It allowed us to investigate the age-specific income gradient in child mental health from early childhood to adolescence, which - to the best of our knowledge - has not been examined in previous longitudinal studies.

Another strength is the consistent cohort across the seven surveys of the MCS, which included repeated measures of income and mental health, allowing us to compare the association between income and child mental health over time on the same sample of children. Restricting the sample to children for which the person completing the survey was always the biological mother further improved consistency in reporting. Using measures of SDQ Total Difficulties Score, internalising and externalising subscales, we were able to compare the income gradient in both overall and distinct aspects of child mental health problems, which may offer different insights for policy and practice aiming at reducing child mental health problems.

One limitation of this study is that the relationship between family income and child mental health cannot be interpreted as a causal relationship. Even though we reduced potential bias caused by time-invariant unobserved heterogeneity, there may be sources of unobserved heterogeneity that are not constant over time [28]. The unobserved time-varying individual effects cannot be captured by the fixed-effects estimation method conducted in this study, which may bias the estimates.

Another limitation is that there might be reporting bias in measures of child mental health. In our study, a significantly higher total difficulties score was observed at 3 years than at other ages, and this was mainly driven by the high externalising problems score at this age (Table 2). This may be explained by the fact that some child behaviours, such as aggressive behaviour, may be captured as externalising problems by SDQ, but these behaviours are normal in preschool children [77]. A potential over-identification of child mental health problems may bias the estimation between income and child mental health at 3 years old, particularly if behavioural expectations or reporting are socially patterned.

Linked to the above issue, SDQ varies among assessors [78, 79], which means that relying on SDQ reported by mothers, as in this study, may identify different children with mental health problems, compared to reports from teachers or children themselves. Within the current research field, it is not clear which of the assessments from teachers, parents or children should be considered the gold standard and it may vary by ages. Presenting results using SDQ from all three assessors would offer a more comprehensive picture. However, the teacher and child-reported SDQ were available only in some surveys of MCS, while mother-reported SDQ was available in all surveys from ages 3 to 17. Therefore, this study did not use teacher and child-reported SDQ, acknowledging this as a limitation of the study. In addition, assessment of a child’s mental health status may be biased if the mother had mental health problems at the time of reporting.

Finally, selection bias due to missing data may affect the generalisability of the findings. While MCS sample weights were applied to adjust for any differential due to loss to follow-up at the 17-year-old survey, they may not fully account for bias introduced by the exclusion of children who did not participant in all survey waves. In addition, the study population was restricted to children whose biological mother answered the SDQ at each survey. Missingness due to this inclusion criteria cannot be adjusted for through the sample weights and multiple imputation used in the main analysis, which may have reduced generalisability. To address this, we conducted a sensitivity analysis extending multiple imputation to the initial MCS sample. This approach accounted for missingness due to item non-response and unit non-response, and cases where the SDQ was not reported by the biological mother. The results were consistent with those of the main analysis, supporting the robustness of our findings. However, the results from the sensitivity analysis should be interpreted with caution, as 32.4% of the study population included in this analysis missed three or more waves, limiting the information available to support accurate imputation for these individuals. Similar results across complete cases and the main analysis with imputed data indicate the limited effect of item nonresponse on findings.

### Policy implications

As mental health problems become increasingly prevalent [80], addressing health inequalities has remained a priority in mental health policy and service delivery within the UK [81]. Previous research has found that income inequalities in child mental health have been increased in the past two decades in the UK [82]. Building on this evidence, this study observes the presence of income inequalities in child mental health among UK children born in the Millennium cohort and highlights the protective effect of family income on child mental health. This may indicate that implementing income-support intervention might be a way to reduce child mental health problems, and children with low income should be the primary targeted population. Monetary interventions such as cash transfer programs and tax and benefit systems are ‘multipurpose’ policies that aim at addressing various social problems simultaneously by changing income which could be relevant to all of them [15]. They may not explicitly be designed to improve child mental health, but they are considered to play an important role in reducing child health inequalities, and may also provide support for the improvement of child mental health [18]. Income interventions such as unconditional household income transfer, Child Benefit programs, and higher Earned Income Tax Credit have lifted families out of poverty and improved children’s mental health in Canada [83] and the US [84, 85]. The economic and political landscape in the UK over the past 20 years has seen considerable change, including an extended period of economic austerity [86, 87]; action now to reduce child poverty will benefit not only physical but also mental health outcomes.

This research found adolescence was a sensitive period when children were more vulnerable if exposed to low income, which means that the same amount of income support would generate greater benefits on child mental health in adolescence than in other child developmental stages. Therefore, public policies targeted at reducing income-related inequalities in child mental health may consider adolescence as an important period for the policies to be implemented. This implication is in line with the finding from the study by Akee, Copeland [85] using longitudinal data from the US, which found a larger positive effect of unconditional household income transfer on child mental health during adolescence than other periods. Adolescence is especially an important period of intervention if the policy targets tackling internalising problems, as this research observed an increased income gradient in internalising problems when children grow older.

## Conclusions

A child mental health-income gradient remains in the UK, even after controlling for known confounders and unobserved heterogeneity. However, the gradient varies with children’s age, being larger in adolescence than in childhood, especially for internalising symptoms. These findings suggest that the timing of policy intervention is important for reducing income-related child mental health inequalities. Income interventions in early adolescence are more likely to reduce mental health symptoms, especially internalising problems. Policies that aim to reduce child mental health problems should pay attention to specific groups that are particularly at risk of exposure factors that adversely impact mental health.

## Electronic Supplementary Material

Below is the link to the electronic supplementary material.

Supplementary Material 1

Supplementary Material 2

Supplementary Material 3

Supplementary Material 4

Supplementary Material 5

Supplementary Material 6

Supplementary Material 7

Supplementary Material 8

Supplementary Material 9

Supplementary Material 10

Supplementary Material 11

Supplementary Material 12

Supplementary Material 13

Supplementary Material 14

Supplementary Material 15

Supplementary Material 16

Supplementary Material 17

Supplementary Material 18

Supplementary Material 19

## Data Availability

The datasets used during the current study are available from the UK Data Service database (https://beta.ukdataservice.ac.uk/datacatalogue/series/series?id=2000031).

## Abbreviation

BMI: Body Mass Index
FE: Fixed-effects
MCS: Millennium Cohort Study
RE: Random-effects
SD: Standard deviation
SDQ: Strengths and Difficulties Questionnaire
SES: Socioeconomic status
TDS: Total Difficulties Score
