# Supplementary material for: The changing role of family income in mental health from childhood to adolescence: findings from a UK longitudinal study
Source: Arch Public Health. 2025 Sep 1;83:224. doi: 10.1186/s13690-025-01702-4 (PMC12400625; doi:10.1186/s13690-025-01702-4)
Supplement: Supplementary file 1 — Supplementary Material 1 [file 13690_2025_1702_MOESM1_ESM.docx]

**Figure A1. Marginal effects of income on internalising/externalising problems**
