# Supplementary material for: The changing role of family income in mental health from childhood to adolescence: findings from a UK longitudinal study
Source: Arch Public Health. 2025 Sep 1;83:224. doi: 10.1186/s13690-025-01702-4 (PMC12400625; doi:10.1186/s13690-025-01702-4)
Supplement: Supplementary file 2 — Supplementary Material 2 [file 13690_2025_1702_MOESM2_ESM.docx]

|  |
| --- |
|  |
|  |

**Figure A2. Marginal effects of income on child mental health problems**

S3 fully-adjusted model used; sample weight used; two curves represent regression results from main analysis (30 imputations, analytical sample of 5,667 children) and sensitivity analysis (70 imputations, 18,294 children).
