# Supplementary material for: The changing role of family income in mental health from childhood to adolescence: findings from a UK longitudinal study
Source: Arch Public Health. 2025 Sep 1;83:224. doi: 10.1186/s13690-025-01702-4 (PMC12400625; doi:10.1186/s13690-025-01702-4)
Supplement: Supplementary file 3 — Supplementary Material 3 [file 13690_2025_1702_MOESM3_ESM.docx]

|  |  |
| --- | --- |

**Figure A3. Marginal effects of income on internalising/externalising problems by child sex**

Sensitivity analysis using 70 imputed datasets, N=18,294; S3 fully-adjusted model used; n=9415 for boys and n=8879 for girls; sample weight used.
