# Supplementary material for: The changing role of family income in mental health from childhood to adolescence: findings from a UK longitudinal study
Source: Arch Public Health. 2025 Sep 1;83:224. doi: 10.1186/s13690-025-01702-4 (PMC12400625; doi:10.1186/s13690-025-01702-4)
Supplement: Supplementary file 4 — Supplementary Material 4 [file 13690_2025_1702_MOESM4_ESM.docx]

|  |
| --- |

**Figure A4. Marginal effects of income on child overall mental health problems**

S3 fully-adjusted model used; N=2,895 for the complete case, N=5,667 for the main analysis; sample weight used.
