# Supplementary material for: The changing role of family income in mental health from childhood to adolescence: findings from a UK longitudinal study
Source: Arch Public Health. 2025 Sep 1;83:224. doi: 10.1186/s13690-025-01702-4 (PMC12400625; doi:10.1186/s13690-025-01702-4)
Supplement: Supplementary file 5 — Supplementary Material 5 [file 13690_2025_1702_MOESM5_ESM.docx]

## Table A1. Variable definition

| Variable | Type | Category | Description |
| --- | --- | --- | --- |
| **Initial endowments** | | | |
| Child age at interview | Continuous | _ | _ |
| Child sex | Binary | 0=Female; 1=Male | _ |
| Child ethnicity | Binary | 0=White; 1= Minority ethnic group | _ |
| Gestational age | Binary | 0=Term (37-41 weeks); 1=Pre-term (<37 weeks) | Excluded post-term infants (42-43 weeks) based on Boyle *et al*.(2012) |
| Firstborn | Binary | 0=Not firstborn 1=firstborn | _ |
| **Confounding factors** | | | |
| **Pregnancy-related factors** | | | |
| Maternal age at childbirth | Categorical | Less than 20 years; 20-24 years; 25-29 years; 30-34 years; 35 years or over | _ |
| Maternal smoking during pregnancy | Categorical | Never smoked; stopped smoking during pregnancy; smoked throughout pregnancy | _ |
| Maternal alcohol consumption during pregnancy | Categorical | Never; light; moderate/heavy | _ |
| Breastfeeding duration | Categorical | Never breastfed; <2 months; 2.0-5.9 months; >=6 months | _ |
| **Child characteristics** | | | |
| Child with limiting physical longstanding illness | Binary | 0=No illness; 1=Had illness | Illness coded by ICD10; excluded mental and behavioural disorders. |
| Child BMI | Categorical | Normal; overweight; obese | Comparing cohort member’s Body Mass Index (BMI) with the International Obesity Task Force (IOTF) thresholds for overweight and obesity. |
| **Family socio-economic characteristics** | | | |
| Lone parent | Binary | 0= two-parent family; 1=lone-parent family | _ |
| Change in family structure | Categorical | No change; New partner; became single | _ |
| Maternal education | Categorical | NVQ Level 1&2; NVQ Level 3; NVQ Level 4&5; none of these | NVQ stands for national vocational qualification; a higher NVQ level indicates a higher educational level. |
| **Wave control** | | | |
| Wave variable | Categorical | 1=Wave 1, 2=Wave 2, …, 7=Wave 7 | This variable was generated to capture the specific survey the observations belonged to. This variable controlled for omitted variables that were individual-invariant but time-varying. |

References:

1. Boyle, E. M., Poulsen, G., Field, D. J., Kurinczuk, J. J., Wolke, D., Alfirevic, Z., & Quigley, M. A. (2012). Effects of gestational age at birth on health outcomes at 3 and 5 years of age: population based cohort study. BMJ, 344.
