# Supplementary material for: The changing role of family income in mental health from childhood to adolescence: findings from a UK longitudinal study
Source: Arch Public Health. 2025 Sep 1;83:224. doi: 10.1186/s13690-025-01702-4 (PMC12400625; doi:10.1186/s13690-025-01702-4)
Supplement: Supplementary file 6 — Supplementary Material 6 [file 13690_2025_1702_MOESM6_ESM.docx]

**Table A2. Summary characteristics of other explanatory variables**

| Variables - n (%) | 3 years | 5 years | 7 years | 11 years | 14 years | 17 years |
| --- | --- | --- | --- | --- | --- | --- |
| **Pregnancy-related factors** |  |  |  |  |  |  |
| Maternal age at childbirth |  |  |  |  |  |  |
| Less than 20 years | 258 (3.7) | - | - | - | - | - |
| 20 to 24 years | 781 (11.2) | - | - | - | - | - |
| 25 to 29 years | 1590 (27.8) | - | - | - | - | - |
| 30 to 34 years | 1951 (36.2) | - | - | - | - | - |
| 35 or over | 1086 (21.1) | - | - | - | - | - |
| Maternal smoking during pregnancy |  |  |  |  |  |  |
| Never smoked | 3991 (71.9) | - | - | - | - | - |
| Stopped smoking during pregnancy | 664 (12.2) | - | - | - | - | - |
| Smoked throughout pregnancy | 1012 (16.0) | - | - | - | - | - |
| Maternal alcohol consumption during pregnancy |  |  |  |  |  |  |
| Never | 3781 (62.7) | - | - | - | - | - |
| Light | 1488 (29.9) | - | - | - | - | - |
| Moderate/Heavy | 397 (7.5) | - | - | - | - | - |
| Breastfeeding |  |  |  |  |  |  |
| Never breastfed | 1448 (22.7) | - | - | - | - | - |
| <2 months | 1486 (25.1) | - | - | - | - | - |
| 2.0-5.9 months | 1160 (21.7) | - | - | - | - | - |
| ≥ 6 months | 1573 (30.5) | - | - | - | - | - |
| **Child characteristics** |  |  |  |  |  |  |
| Child with limiting longstanding illness | 153 (2.7) | 283 (4.6) | 295 (5.1) | 261 (4.5) | 306 (5.3) | 244 (4.3) |
| Child BMI |  |  |  |  |  |  |
| Normal | 4318 (77.0) | 4483 (79.8) | 4539 (81.1) | 4114 (74.0) | 4137 (74.3) | 3950 (71.0) |
| Overweight | 1020 (17.5) | 895 (15.4) | 810 (13.8) | 1190 (20.3) | 1128 (18.9) | 1099 (19.1) |
| Obese | 329 (5.5) | 289 (4.8) | 318 (5.1) | 363 (5.7) | 402 (6.8) | 618 (9.9) |
| **Family socio-economic characteristics** | | | | | | |
| Lone parent | 720 (11.1) | 873 (13.9) | 963 (15.4) | 1156 (19.9) | 1235 (21.1) | 1519 (26.2) |
| Change in family structure |  |  |  |  |  |  |
| No change | 5151 (92.5) | 5214 (92.0) | 5236 (92.4) | 4998 (88.1) | 5151 (90.8) | 5344 (94.3) |
| New partner | 227 (2.9) | 176 (3.0) | 176 (3.2) | 272 (4.4) | 227 (4.1) | 28 (0.5) |
| Became single | 289 (4.6) | 277 (5.0) | 255 (4.4) | 397 (7.5) | 289 (5.1) | 295 (5.2) |
| Maternal education |  |  |  |  |  |  |
| NVQ Level 1&2 | 1887 (34.0) | 1887 (34.0) | 1779 (32.0) | 1604 (29.0) | 1507 (27.2) | 1507 (27.2) |
| NVQ Level 3 | 861 (14.6) | 861 (14.6) | 890 (15.2) | 856 (14.6) | 839 (14.2) | 839 (14.2) |
| NVQ Level 4&5 | 2346 (43.8) | 2346 (43.8) | 2476 (45.9) | 2743 (50.4) | 2890 (53.0) | 2890 (53.0) |
| None of these | 573 (7.6) | 573 (7.6) | 522 (6.9) | 464 (6.0) | 431 (5.6) | 431 (5.6) |
| Notes: N=5667; unweighted counts (n) and survey-weighted mean, standard deviation (SD) and proportions (%) reported; - baseline variable which remains the same across all surveys. | | | | | | |
