# Supplementary material for: The changing role of family income in mental health from childhood to adolescence: findings from a UK longitudinal study
Source: Arch Public Health. 2025 Sep 1;83:224. doi: 10.1186/s13690-025-01702-4 (PMC12400625; doi:10.1186/s13690-025-01702-4)
Supplement: Supplementary file 7 — Supplementary Material 7 [file 13690_2025_1702_MOESM7_ESM.docx]

## Table A3 Baseline characteristics of complete cases vs. incomplete cases (3 years old)

| Characteristic | Complete cases (n=2895) | Incomplete cases (n=2772) | *p*-value |
| --- | --- | --- | --- |
| Overall mental health problems (Mean) | 8.5 | 9.6 | <0.001 |
| Internalising problems (Mean) | 2.5 | 2.9 | <0.001 |
| Externalising problems (Mean) | 6.0 | 6.8 | <0.001 |
| Lagged transitory income (£) (Mean) | 30325 | 25653 | <0.001 |
| Child in poverty at 9 months (%) | 17.7 | 33.0 | <0.001 |
| Child age, years (mean) | 3.10 | 3.13 | <0.001 |
| Male child (%) | 47.6 | 49.0 | 0.307 |
| Ethnic minority child (%) | 7.4 | 17.6 | <0.001 |
| Firstborn (%) | 50.0 | 52.1 | 0.112 |
| Pre-term (%) | 6.7 | 7.2 | 0.471 |
| Maternal age (%) |  |  | <0.001 |
| Less than 20 | 3.5 | 5.6 |  |
| 20 to 24 years | 11.6 | 16.0 |  |
| 25-29 years | 27.6 | 28.5 |  |
| 30 to 34 years | 37.0 | 31.8 |  |
| 35 or over | 20.3 | 18.1 |  |
| Maternal smoking during pregnancy (%) |  |  | <0.001 |
| Never smoked | 72.9 | 67.9 |  |
| Stopped smoking during pregnancy | 11.6 | 11.9 |  |
| Smoked throughout pregnancy | 15.5 | 20.2 |  |
| Maternal alcohol consumption during pregnancy (%) | |  | <0.001 |
| Never | 64.3 | 69.3 |  |
| Light | 28.7 | 23.7 |  |
| Moderate/Heavy | 7.0 | 7.0 |  |
| Breastfeeding (%) |  |  | 0.001 |
| Never breastfed | 23.4 | 27.8 |  |
| <2 months | 26.3 | 26.2 |  |
| 2.0-5.9 months | 21.1 | 19.8 |  |
| ≥ 6 months | 29.2 | 26.2 |  |
| Child with limiting physical longstanding illness (%) | 2.4 | 3.1 | 0.108 |
| Child BMI (%) |  |  | 0.081 |
| Normal | 76.6 | 75.9 |  |
| Overweight | 18.2 | 17.5 |  |
| Obese | 5.2 | 6.6 |  |
| Lone parent (%) | 9.9 | 15.6 | <0.001 |
| Change in family structure (%) |  |  | <0.001 |
| No change | 92.5 | 89.4 |  |
| New partner | 3.1 | 5.0 |  |
| Became single | 4.4 | 5.6 |  |
| Maternal education (%) |  |  | <0.001 |
| NVQ Level 1&2 | 31.3 | 35.4 |  |
| NVQ Level 3 | 16.8 | 13.6 |  |
| NVQ Level 4&5 | 46.3 | 36.3 |  |
| None of these | 5.6 | 14.7 |  |
