# Supplementary material for: The changing role of family income in mental health from childhood to adolescence: findings from a UK longitudinal study
Source: Arch Public Health. 2025 Sep 1;83:224. doi: 10.1186/s13690-025-01702-4 (PMC12400625; doi:10.1186/s13690-025-01702-4)
Supplement: Supplementary file 8 — Supplementary Material 8 [file 13690_2025_1702_MOESM8_ESM.docx]

## Table A4. Hausman test

|  | FE coef. | RE coef. | Difference | Sqrt(diag(V_b-V_B)) Standard Error |
| --- | --- | --- | --- | --- |
| Variable | b | B | b-B |  |
| Lagged transitory income | -0.040 | -0.085 | 0.044 | 0.004 |
| Survey wave (child age) |  |  |  |  |
| Wave 2 (3 years) # | - | - | - | - |
| Wave 3 (5 years) | -0.804 | -0.799 | -0.005 | 0.001 |
| Wave 4 (7 years) | -0.797 | -0.723 | -0.074 | 0.001 |
| Wave 5 (11 years) | -0.426 | -0.309 | -0.117 | 0.017 |
| Wave 6 (14 years) | 0.151 | 0.539 | -0.388 | 0.035 |
| Wave 7 (17 years) | -0.509 | -0.161 | -0.347 | 0.036 |
| Income and wave interaction |  |  |  |  |
| Income × Wave 2 # | - | - | - | - |
| Income × Wave 3 | 0.040 | 0.039 | 0.001 | 0.000 |
| Income × Wave 4 | 0.041 | 0.034 | 0.007 | 0.001 |
| Income × Wave 5 | 0.008 | -0.003 | 0.011 | 0.002 |
| Income × Wave 6 | -0.043 | -0.080 | 0.037 | 0.003 |
| Income × Wave 7 | 0.013 | -0.021 | 0.034 | 0.005 |
| Child characteristics |  |  |  |  |
| Child with longstanding illness | 0.087 | 0.133 | -0.046 | 0.004 |
| Child BMI |  |  |  |  |
| Normal # | - | - | - | - |
| Overweight | 0.036 | 0.045 | -0.001 | 0.009 |
| Obese | 0.123 | 0.150 | -0.026 | 0.009 |
| Family characteristics |  |  |  |  |
| Lone parent | 0.082 | 0.108 | -0.025 | 0.005 |
| Change in family structure |  |  |  |  |
| No change # | - | - | - | - |
| New partner | 0.041 | 0.062 | -0.020 | 0.005 |
| Became single | -0.037 | -0.030 | -0.008 | 0.005 |
| Maternal education |  |  |  |  |
| NVQ Level 1&2 # | - | - | - | - |
| NVQ Level 3 | 0.030 | -0.058 | 0.087 | 0.021 |
| NVQ Level 4&5 | 0.058 | -0.106 | 0.163 | 0.021 |
| None of these | 0.286 | 0.278 | 0.008 | 0.037 |
| **Chi-square** | **301.11** |  |  |  |
| **p-value** | **<0.0001** |  |  |  |

Notes: TDS as the dependent variable; FE fixed-effects, RE random-effects; # reference category; N=5667.
