# Supplementary material for: The changing role of family income in mental health from childhood to adolescence: findings from a UK longitudinal study
Source: Arch Public Health. 2025 Sep 1;83:224. doi: 10.1186/s13690-025-01702-4 (PMC12400625; doi:10.1186/s13690-025-01702-4)
Supplement: Supplementary file 9 — Supplementary Material 9 [file 13690_2025_1702_MOESM9_ESM.docx]

## Table A5. The association between family income and child overall mental health problems (TDS)

|  | S1 | S2 |
| --- | --- | --- |
| Lagged transitory income | -0.026 | -0.017 |
|  | (0.027) | (0.027) |
| Survey wave (child age) | | |
| Wave 2 (3 years) # | - | - |
| Wave 3 (5 years) | -0.760*** | -0.763*** |
|  | (0.240) | (0.241) |
| Wave 4 (7 years) | -0.560** | -0.567** |
|  | (0.238) | (0.239) |
| Wave 5 (11 years) | 0.046 | 0.036 |
|  | (0.275) | (0.276) |
| Wave 6 (14 years) | 0.812* | 0.780* |
|  | (0.419) | (0.430) |
| Wave 7 (17 years) | 0.137 | 0.015 |
|  | (0.442) | (0.443) |
| Income and wave interaction | | |
| Income × Wave 2 # | - | - |
| Income × Wave 3 | 0.035 | 0.035 |
|  | (0.024) | (0.024) |
| Income × Wave 4 | 0.018 | 0.019 |
|  | (0.023) | (0.023) |
| Income × Wave 5 | -0.036 | -0.036 |
|  | (0.027) | (0.027) |
| Income × Wave 6 | -0.105** | -0.103** |
|  | (0.041) | (0.042) |
| Income × Wave 7 | -0.049 | -0.038 |
|  | (0.043) | (0.043) |
| Child characteristics |  |  |
| Child with longstanding illness |  | 0.091*** |
|  |  | (0.028) |
| Child BMI |  |  |
| Normal # |  | - |
| Overweight |  | 0.047*** |
|  |  | (0.017) |
| Obese |  | 0.135*** |
|  |  | (0.032) |
| Family characteristics |  |  |
| Lone parent |  | 0.064* |
|  |  | (0.033) |
| Change in family structure |  |  |
| No change # |  | - |
| New partner |  | 0.033 |
|  |  | (0.030) |
| Became single |  | -0.026 |
|  |  | (0.027) |
| Maternal education |  |  |
| NVQ Level 1&2 # |  | - |
| NVQ Level 3 |  | 0.014 |
|  |  | (0.043) |
| NVQ Level 4&5 |  | 0.017 |
|  |  | (0.043) |
| None of these |  | 0.289*** |
|  |  | (0.073) |

Notes: S1=raw regression, S2=S1+wave+interaction, S3=S2+covariates, fully-adjusted model; N=5667; # reference category; * *p*<0.1 ** *p*<0.05 ****p*<0.001; standard errors in parentheses; sample weights used.
