# Supplementary material for: The changing role of family income in mental health from childhood to adolescence: findings from a UK longitudinal study
Source: Arch Public Health. 2025 Sep 1;83:224. doi: 10.1186/s13690-025-01702-4 (PMC12400625; doi:10.1186/s13690-025-01702-4)
Supplement: Supplementary file 10 — Supplementary Material 10 [file 13690_2025_1702_MOESM10_ESM.docx]

## Table A6. Marginal effects of income on child overall mental health problems

| Age | S1 | S2 |
| --- | --- | --- |
| 3 | -0.026 | -0.017 |
|  | (0.027) | (0.027) |
| 5 | 0.009 | 0.018 |
|  | (0.018) | (0.017) |
| 7 | -0.008 | 0.001 |
|  | (0.020) | (0.020) |
| 11 | -0.062*** | -0.053** |
|  | (0.022) | (0.022) |
| 14 | -0.131*** | -0.120*** |
|  | (0.034) | (0.036) |
| 17 | -0.074** | -0.055 |
|  | (0.036) | (0.036) |

Notes: S1 baseline model, controls for wave and income and wave interaction, S2 fully adjusted model further controls for initial endowments and confounders; N=5667; * *p*<0.1 ** *p*<0.05 ****p*<0.001; standard errors in parentheses; sample weights used.
