# Supplementary material for: The changing role of family income in mental health from childhood to adolescence: findings from a UK longitudinal study
Source: Arch Public Health. 2025 Sep 1;83:224. doi: 10.1186/s13690-025-01702-4 (PMC12400625; doi:10.1186/s13690-025-01702-4)
Supplement: Supplementary file 11 — Supplementary Material 11 [file 13690_2025_1702_MOESM11_ESM.docx]

**Table A7. The association between family income and internalising problems**

|  | S1 | S2 |  |
| --- | --- | --- | --- |
| Lagged transitory income | 0.029 | 0.031 |  |
|  | (0.028) | (0.028) |  |
| Survey wave (child age) |  |  |  |
| Wave 2 (3 years) # | - | - |  |
| Wave 3 (5 years) | -0.273 | -0.286 |  |
|  | (0.227) | (0.229) |  |
| Wave 4 (7 years) | 0.046 | 0.026 |  |
|  | (0.241) | (0.243) |  |
| Wave 5 (11 years) | 0.953*** | 0.883*** |  |
|  | (0.299) | (0.298) |  |
| Wave 6 (14 years) | 2.163*** | 2.075*** |  |
|  | (0.471) | (0.474) |  |
| Wave 7 (17 years) | 2.441*** | 2.200*** |  |
|  | (0.494) | (0.491) |  |
| Income and wave interaction | | | |
| Income × Wave 2 # | - | - |  |
| Income × Wave 3 | 0.016 | 0.017 |  |
|  | (0.022) | (0.022) |  |
| Income × Wave 4 | -0.008 | -0.006 |  |
|  | (0.024) | (0.024) |  |
| Income × Wave 5 | -0.080*** | -0.074** |  |
|  | (0.029) | (0.029) |  |
| Income × Wave 6 | -0.182*** | -0.174*** |  |
|  | (0.046) | (0.046) |  |
| Income × Wave 7 | -0.206*** | -0.185*** |  |
|  | (0.048) | (0.048) |  |
| Child characteristics |  |  |  |
| Child longstanding illness |  | 0.116*** |  |
|  |  | (0.036) |  |
| Child BMI |  |  |  |
| Normal # |  | - |  |
| Overweight |  | 0.087*** |  |
|  |  | (0.021) |  |
| Obese |  | 0.218*** |  |
|  |  | (0.040) |  |
| Family characteristics |  |  |  |
| Lone parent |  | 0.076*** |  |
|  |  | (0.028) |  |
| Maternal education |  |  |  |
| NVQ Level 1&2 # |  | - |  |
| NVQ Level 3 |  | 0.048 |  |
|  |  | (0.052) |  |
| NVQ Level 4&5 |  | 0.055 |  |
|  |  | (0.048) |  |
| None of these |  | 0.250*** |  |
|  |  | (0.084) |  |
|  |  |  |  |

Notes: S1 baseline model controls for wave and income and wave interaction, S3 fully adjusted model; N=5667; # reference category; * *p*<0.1 ** *p*<0.05 ****p*<0.001; standard errors in parentheses; sample weights used.
