# Supplementary material for: The changing role of family income in mental health from childhood to adolescence: findings from a UK longitudinal study
Source: Arch Public Health. 2025 Sep 1;83:224. doi: 10.1186/s13690-025-01702-4 (PMC12400625; doi:10.1186/s13690-025-01702-4)
Supplement: Supplementary file 12 — Supplementary Material 12 [file 13690_2025_1702_MOESM12_ESM.docx]

**Table A8. The association between family income and externalising problems**

|  | | S1 | S2 |
| --- | --- | --- | --- |
| Lagged transitory income | | -0.064*** | -0.059** |
|  | | (0.025) | (0.025) |
| Survey wave (child age) | |  |  |
| Wave 2 (3 years) # | | - | - |
| Wave 3 (5 years) | | -0.937*** | -0.936*** |
|  | | (0.254) | (0.254) |
| Wave 4 (7 years) | | -0.900*** | -0.882*** |
|  | | (0.253) | (0.253) |
| Wave 5 (11 years) | | -0.736*** | -0.671** |
|  | | (0.261) | (0.261) |
| Wave 6 (14 years) | | -0.581 | -0.455 |
|  | | (0.371) | (0.372) |
| Wave 7 (17 years) | | -1.837*** | -1.725*** |
|  | | (0.384) | (0.384) |
|  |  | | |
| Income × Wave 2 # | | - | - |
| Income × Wave 3 | | 0.040 | 0.040 |
|  | | (0.025) | (0.025) |
| Income × Wave 4 | | 0.034 | 0.033 |
|  | | (0.025) | (0.025) |
| Income × Wave 5 | | 0.012 | 0.006 |
|  | | (0.025) | (0.025) |
| Income × Wave 6 | | -0.008 | -0.020 |
|  | | (0.036) | (0.036) |
| Income × Wave 7 | | 0.099*** | 0.088** |
|  | | (0.038) | (0.038) |
| Child characteristics | |  |  |
| Child with physical longstanding illness | |  | 0.042* |
|  | |  | (0.025) |
| Family characteristics | |  |  |
| Maternal education | |  |  |
| NVQ Level 1&2 # | |  | - |
| NVQ Level 3 | |  | -0.020 |
|  | |  | (0.043) |
| NVQ Level 4&5 | |  | -0.019 |
|  | |  | (0.042) |
| None of these | |  | 0.232*** |
|  | |  | (0.075) |

Notes: S1, baseline model controls for wave and income and wave interaction, S3 fully adjusted model; N=5667; # reference category; * *p*<0.1 ** *p*<0.05 ****p*<0.001; standard errors in parentheses; sample weights used.
