# Supplementary material for: The changing role of family income in mental health from childhood to adolescence: findings from a UK longitudinal study
Source: Arch Public Health. 2025 Sep 1;83:224. doi: 10.1186/s13690-025-01702-4 (PMC12400625; doi:10.1186/s13690-025-01702-4)
Supplement: Supplementary file 13 — Supplementary Material 13 [file 13690_2025_1702_MOESM13_ESM.docx]

**Table A9. Marginal effects of income on child internalising and externalising problems**

|  | Internalising | | Externalising | |
| --- | --- | --- | --- | --- |
| Age | S1 | S2 | S1 | S2 |
| 3 | 0.029 | 0.031 | -0.064*** | -0.059** |
|  | (0.028) | (0.028) | (0.025) | (0.025) |
| 5 | 0.045** | 0.048** | -0.024 | -0.020 |
|  | (0.021) | (0.021) | (0.016) | (0.016) |
| 7 | 0.021 | 0.025 | -0.029 | -0.026 |
|  | (0.022) | (0.022) | (0.019) | (0.020) |
| 11 | -0.051** | -0.043* | -0.052*** | -0.053*** |
|  | (0.024) | (0.024) | (0.020) | (0.020) |
| 14 | -0.153*** | -0.143*** | -0.072** | -0.079** |
|  | (0.041) | (0.041) | (0.031) | (0.031) |
| 17 | -0.178*** | -0.154*** | 0.035 | 0.029 |
|  | (0.043) | (0.042) | (0.032) | (0.032) |

Notes: S1 baseline model controls for wave and income and wave interaction, S2 is fully-adjusted model; N=5667; * *p*<0.1 ** *p*<0.05 ****p*<0.001; standard errors in parentheses; sample weights used; lagged transitory income used.
