# Supplementary material for: The changing role of family income in mental health from childhood to adolescence: findings from a UK longitudinal study
Source: Arch Public Health. 2025 Sep 1;83:224. doi: 10.1186/s13690-025-01702-4 (PMC12400625; doi:10.1186/s13690-025-01702-4)
Supplement: Supplementary file 14 — Supplementary Material 14 [file 13690_2025_1702_MOESM14_ESM.docx]

**Table A10. Marginal effects of poverty on child overall mental health problems**

| Age | TDS | |
| --- | --- | --- |
|  | S1 | S2 |
| 3 | 0.011 | 0.002 |
|  | (0.040) | (0.039) |
| 5 | -0.042 | -0.036 |
|  | (0.031) | (0.031) |
| 7 | -0.026 | -0.019 |
|  | (0.027) | (0.027) |
| 11 | 0.048 | 0.059* |
|  | (0.031) | (0.031) |
| 14 | 0.104** | 0.096** |
|  | (0.045) | (0.045) |
| 17 | 0.061 | 0.062 |
|  | (0.043) | (0.043) |

Notes: S1 controls for wave and income and wave interaction, S2 is fully-adjusted model; N=5667; * *p*<0.1 ** *p*<0.05 ****p*<0.001; standard errors in parentheses; sample weights used; lagged transitory poverty used.
