# Supplementary material for: The changing role of family income in mental health from childhood to adolescence: findings from a UK longitudinal study
Source: Arch Public Health. 2025 Sep 1;83:224. doi: 10.1186/s13690-025-01702-4 (PMC12400625; doi:10.1186/s13690-025-01702-4)
Supplement: Supplementary file 15 — Supplementary Material 15 [file 13690_2025_1702_MOESM15_ESM.docx]

**Table A11. The association between poverty and child overall mental health problems (TDS)**

|  | | S1 | | S2 | | |
| --- | --- | --- | --- | --- | --- | --- |
| Lagged transitory poverty | | 0.023 | | 0.011 | | |
|  | | (0.040) | | (0.040) | | |
| Survey wave (child age) | |  | |  | | |
| Wave 2 (3 years) # | | - | | - | | |
| Wave 3 (5 years) | | -0.397*** | | -0.398*** | | |
|  | | (0.014) | | (0.014) | | |
| Wave 4 (7 years) | | -0.370*** | | -0.372*** | | |
|  | | (0.015) | | (0.016) | | |
| Wave 5 (11 years) | | -0.323*** | | -0.331*** | | |
|  | | (0.017) | | (0.018) | | |
| Wave 6 (14 years) | | -0.269*** | | -0.280*** | | |
|  | | (0.020) | | (0.021) | | |
| Wave 7 (17 years) | | -0.361*** | | -0.376*** | | |
|  | | (0.019) | | (0.021) | | |
| Poverty and wave interaction | |  | |  | | |
| No × Wave 2 # | | - | | - | | |
| No × Wave 3 # | | - | | - | | |
| No × Wave 4 # | | - | | - | | |
| No × Wave 5 # | | - | | - | | |
| No × Wave 6 # | | - | | - | | |
| No × Wave 7 # | | - | | - | | |
| Yes × Wave 2 # | | - | | - | | |
| Yes × Wave 3 | | -0.048 | | -0.053 | | |
|  | | (0.038) | | (0.038) | | |
| Yes × Wave 4 | | -0.037 | | -0.037 | | |
|  | | (0.041) | | (0.041) | | |
| Yes × Wave 5 | | 0.035 | | 0.036 | | |
|  | | (0.041) | | (0.041) | | |
| Yes × Wave 6 | | 0.087 | | 0.093 | | |
|  | | (0.059) | | (0.060) | | |
| Yes × Wave 7 | | 0.052 | | 0.049 | | |
|  | | (0.055) | | (0.055) | | |
| Child characteristics | |  | |  | | |
| Child with physical longstanding illness | | |  | | 0.091*** |  |
|  | | |  | | (0.028) |  |
| Child BMI | |  | |  | | |
| Normal# | |  | | - | | |
| Overweight | |  | | 0.048*** | | |
|  | |  | | (0.017) | | |
| Obese | |  | | 0.137*** | | |
|  | |  | | (0.032) | | |
| Family characteristics | |  | |  | | |
| Lone parent | |  | | 0.075** | | |
|  | |  | | (0.032) | | |
| Change in family structure |  |  |  |  |  |  |
| No change # | |  | | - | | |
| New partner | |  | | 0.043 | | |
|  | |  | | (0.030) | | |
| Became single | |  | | -0.038 | | |
|  | |  | | (0.027) | | |
| Maternal education | |  | |  | | |
| NVQ Level 1&2 # | |  | | - | | |
| NVQ Level 3 | |  | | 0.021 | | |
|  | |  | | (0.042) | | |
| NVQ Level 4&5 | |  | | 0.023 | | |
|  | |  | | (0.043) | | |
| None of these | |  | | 0.287*** | | |
|  | |  | | (0.074) | | |

Notes: S1 baseline mode, S2 fully-adjusted model; N=5667; # reference category; * *p*<0.1 ** *p*<0.05 ****p*<0.001; standard errors in parentheses; sample weights used.
