# Supplementary material for: The changing role of family income in mental health from childhood to adolescence: findings from a UK longitudinal study
Source: Arch Public Health. 2025 Sep 1;83:224. doi: 10.1186/s13690-025-01702-4 (PMC12400625; doi:10.1186/s13690-025-01702-4)
Supplement: Supplementary file 16 — Supplementary Material 16 [file 13690_2025_1702_MOESM16_ESM.docx]

**Table A12. Marginal effects of poverty on child internalising and externalising problems**

|  | Internalising | | Externalising | |
| --- | --- | --- | --- | --- |
| Age | S1 | S2 | S1 | S2 |
| 3 | -0.018 | -0.021 | 0.050 | 0.044 |
|  | (0.041) | (0.041) | (0.040) | (0.040) |
| 5 | -0.063* | -0.070* | 0.013 | 0.004 |
|  | (0.037) | (0.036) | (0.029) | (0.028) |
| 7 | -0.021 | -0.025 | -0.004 | -0.008 |
|  | (0.029) | (0.028) | (0.028) | (0.028) |
| 11 | 0.060 | 0.053 | 0.037 | 0.039 |
|  | (0.037) | (0.037) | (0.029) | (0.029) |
| 14 | 0.147*** | 0.142*** | 0.044 | 0.052 |
|  | (0.049) | (0.049) | (0.042) | (0.041) |
| 17 | 0.146*** | 0.130*** | -0.008 | -0.003 |
|  | (0.048) | (0.048) | (0.038) | (0.038) |

Notes: S1 controls for wave and income and wave interaction, S2=fully-adjusted model; N=5667; * *p*<0.1 ** *p*<0.05 ****p*<0.001; standard errors in parentheses; sample weights used.
