# Supplementary material for: The changing role of family income in mental health from childhood to adolescence: findings from a UK longitudinal study
Source: Arch Public Health. 2025 Sep 1;83:224. doi: 10.1186/s13690-025-01702-4 (PMC12400625; doi:10.1186/s13690-025-01702-4)
Supplement: Supplementary file 17 — Supplementary Material 17 [file 13690_2025_1702_MOESM17_ESM.docx]

**Table A13. The association between poverty and internalising problems**

|  | S1 | S2 | |
| --- | --- | --- | --- |
| Lagged transitory poverty | -0.018 | -0.021 | |
|  | (0.041) | (0.041) | |
| Survey wave (child age) |  |  | |
| Wave 2 (3 years) # | - | - | |
| Wave 3 (5 years) | -0.101*** | -0.102*** | |
|  | (0.014) | (0.014) | |
| Wave 4 (7 years) | -0.036** | -0.038** | |
|  | (0.016) | (0.016) | |
| Wave 5 (11 years) | 0.133*** | 0.120*** | |
|  | (0.019) | (0.019) | |
| Wave 6 (14 years) | 0.294*** | 0.277*** | |
|  | (0.020) | (0.020) | |
| Wave 7 (17 years) | 0.326*** | 0.300*** | |
|  | (0.022) | (0.022) | |
| Poverty and wave interaction | | |  |
| No × Wave 2 # | - | - | |
| No × Wave 3 # | - | - | |
| No × Wave 4 # | - | - | |
| No × Wave 5 # | - | - | |
| No × Wave 6 # | - | - | |
| No × Wave 7 # | - | - | |
| Yes × Wave 2 # | - | - | |
| Yes × Wave 3 | -0.045 | -0.050 | |
|  | (0.038) | (0.038) | |
| Yes × Wave 4 | -0.003 | -0.004 | |
|  | (0.041) | (0.041) | |
| Yes × Wave 5 | 0.078* | 0.074 | |
|  | (0.047) | (0.046) | |
| Yes × Wave 6 | 0.165** | 0.163** | |
|  | (0.064) | (0.064) | |
| Yes × Wave 7 | 0.164*** | 0.150** | |
|  | (0.061) | (0.060) | |
| Child characteristics |  |  | |
| Child with physical longstanding illness |  | 0.115*** | |
|  |  | (0.036) | |
| Child BMI |  |  | |
| Normal# |  | - | |
| Overweight |  | 0.091*** | |
|  |  | (0.021) | |
| Obese |  | 0.226*** | |
|  |  | (0.040) | |
| Family characteristics |  |  | |
| Lone parent |  | 0.083*** | |
|  |  | (0.028) | |
| Maternal education |  |  | |
| NVQ Level 1&2 # |  | - | |
| NVQ Level 3 |  | 0.063 | |
|  |  | (0.051) | |
| NVQ Level 4&5 |  | 0.071 | |
|  |  | (0.047) | |
| None of these |  | 0.234*** | |
|  |  | (0.085) | |

Notes: S1 baseline model, S2 fully-adjusted model; N=5667; # reference category; * *p*<0.1 ** *p*<0.05 ****p*<0.001; standard errors in parentheses; sample weights used.
