# Supplementary material for: The changing role of family income in mental health from childhood to adolescence: findings from a UK longitudinal study
Source: Arch Public Health. 2025 Sep 1;83:224. doi: 10.1186/s13690-025-01702-4 (PMC12400625; doi:10.1186/s13690-025-01702-4)
Supplement: Supplementary file 18 — Supplementary Material 18 [file 13690_2025_1702_MOESM18_ESM.docx]

**Table A14. The association between poverty and externalising problems**

|  | | S1 | S2 |
| --- | --- | --- | --- |
| Lagged transitory poverty | | 0.050 | 0.044 |
|  | | (0.040) | (0.040) |
| Survey wave (child age) | |  |  |
| Wave 2 (3 years) # | | - | - |
| Wave 3 (5 years) | | -0.524*** | -0.524*** |
|  | | (0.015) | (0.015) |
| Wave 4 (7 years) | | -0.538*** | -0.537*** |
|  | | (0.017) | (0.017) |
| Wave 5 (11 years) | | -0.608*** | -0.606*** |
|  | | (0.017) | (0.017) |
| Wave 6 (14 years) | | -0.661*** | -0.658*** |
|  | | (0.020) | (0.020) |
| Wave 7 (17 years) | | -0.826*** | -0.822*** |
|  | | (0.019) | (0.019) |
|  |  | | |
| No × Wave 2 # | | - | - |
| No × Wave 3 # | | - | - |
| No × Wave 4 # | | - | - |
| No × Wave 5 # | | - | - |
| No × Wave 6 # | | - | - |
| No × Wave 7 # | | - | - |
| Yes × Wave 2 # | | - | - |
| Yes × Wave 3 | | -0.037 | -0.039 |
|  | | (0.043) | (0.042) |
| Yes × Wave 4 | | -0.054 | -0.052 |
|  | | (0.043) | (0.043) |
| Yes × Wave 5 | | -0.013 | -0.005 |
|  | | (0.041) | (0.041) |
| Yes × Wave 6 | | -0.006 | 0.008 |
|  | | (0.054) | (0.054) |
| Yes × Wave 7 | | -0.058 | -0.047 |
|  | | (0.050) | (0.050) |
| Child characteristics | |  |  |
| Child with physical longstanding illness | |  | 0.042* |
|  | |  | (0.024) |
| Family characteristics | |  |  |
| Maternal education | |  |  |
| NVQ Level 1&2 # | |  | - |
| NVQ Level 3 | |  | -0.023 |
|  | |  | (0.042) |
| NVQ Level 4&5 | |  | -0.022 |
|  | |  | (0.042) |
| None of these | |  | 0.241*** |
|  | |  | (0.076) |

Notes: S1 baseline model, S2 fully-adjusted model; N=5667; # reference category; * *p*<0.1 ** *p*<0.05 ****p*<0.001; standard errors in parentheses; sample weights used.
