# Supplementary material for: The changing role of family income in mental health from childhood to adolescence: findings from a UK longitudinal study
Source: Arch Public Health. 2025 Sep 1;83:224. doi: 10.1186/s13690-025-01702-4 (PMC12400625; doi:10.1186/s13690-025-01702-4)
Supplement: Supplementary file 19 — Supplementary Material 19 [file 13690_2025_1702_MOESM19_ESM.docx]

**Table A15. Comparison of marginal effects of income on child mental health by child sex**

|  | Internalising problems | | | Externalising problems | | |
| --- | --- | --- | --- | --- | --- | --- |
| Age | Girls | Boys | p value | Girls | Boys | p value |
| 3 | 0.029 | 0.026 | 0.944 | -0.062** | -0.057 | 0.908 |
|  | (0.036) | (0.037) |  | (0.031) | (0.036) |  |
| 5 | 0.034 | 0.061** | 0.524 | -0.015 | -0.026 | 0.747 |
|  | (0.031) | (0.028) |  | (0.020) | (0.027) |  |
| 7 | 0.065** | -0.025 | 0.027 | -0.016 | -0.043 | 0.501 |
|  | (0.026) | (0.031) |  | (0.026) | (0.031) |  |
| 11 | -0.070* | -0.017 | 0.268 | -0.032 | -0.077** | 0.250 |
|  | (0.036) | (0.031) |  | (0.025) | (0.030) |  |
| 14 | -0.145*** | -0.140** | 0.950 | -0.064* | -0.087* | 0.712 |
|  | (0.053) | (0.058) |  | (0.038) | (0.052) |  |
| 17 | -0.226*** | -0.073 | 0.067 | 0.034 | 0.038 | 0.944 |
|  | (0.058) | (0.060) |  | (0.039) | (0.052) |  |

Notes: fully-adjusted model used; n=2930 for girls and n=2737 for boys; *p* value obtained from a two-sided z-test testing the difference in the marginal effects of income on child mental health problems between boys and girls; standard errors in parentheses; sample weights used.
